# Etoposide Triggers Cellular Senescence by Inducing Multiple Centrosomes and Primary Cilia in Adrenocortical Tumor Cells

**DOI:** 10.3390/cells10061466

**Published:** 2021-06-11

**Authors:** Yen-Ni Teng, Huei-Cih Chang, Yu-Ying Chao, Hui-Ling Cheng, Wei-Chih Lien, Chia-Yih Wang

**Affiliations:** 1 Department of Biological Sciences and Technology, National University of Tainan, Tainan 700, Taiwan; tengyenni1968@gmail.com (Y.-N.T.); air840805@gmail.com (H.-C.C.); 2 Department of Physical Medicine and Rehabilitation, College of Medicine, National Cheng Kung University, Tainan 701, Taiwan; 3 Department of Cell Biology and Anatomy, College of Medicine, National Cheng Kung University, Tainan 701, Taiwan; yuyingchaoo@gmail.com (Y.-Y.C.); tomato4329@gmail.com (H.-L.C.); 4 Institute of Basic Medical Sciences, College of Medicine, National Cheng Kung University, Tainan 701, Taiwan; 5 Department of Physical Medicine and Rehabilitation, National Cheng Kung University Hospital, College of Medicine, National Cheng Kung University, Tainan 704, Taiwan

**Keywords:** etoposide, centrosome, primary cilia, senescence, autophagy, DNA-PK, Chk2

## Abstract

Etoposide (ETO) has been used in treating adrenocortical tumor (ACT) cells. Our previous study showed that ETO inhibits ACT cell growth. In the present study, we show that ETO treatment at IC50 (10 μM) inhibited ACT cell growth by inducing cellular senescence rather than apoptosis. Several markers of cellular senescence, including enlarged nuclei, activated senescence-associated β-galactosidase activity, elevated levels of p53 and p21, and down-regulation of Lamin B1, were observed. We further found that ETO induced multiple centrosomes. The inhibition of multiple centrosomes accomplished by treating cells with either roscovitine or centrinone or through the overexpression of NR5A1/SF-1 alleviated ETO-induced senescence, suggesting that ETO triggered senescence via multiple centrosomes. Primary cilia also played a role in ETO-induced senescence. In the mechanism, DNA-PK-Chk2 signaling was activated by ETO treatment; inhibition of this signaling cascade alleviated multiple ETO-induced centrosomes and primary cilia followed by reducing cellular senescence. In addition to DNA damage signaling, autophagy was also triggered by ETO treatment for centrosomal events and senescence. Importantly, the inactivation of DNA-PK-Chk2 signaling reduced ETO-triggered autophagy; however, the inhibition of autophagy did not affect DNA-PK-Chk2 activation. Thus, ETO activated the DNA-PK-Chk2 cascade to facilitate autophagy. The activated autophagy further induced multiple centrosomes and primary cilia followed by triggering senescence.

## 1. Introduction

In response to adrenocorticotropic hormone stimulation, the adrenocortex synthesizes and secretes steroid hormones to maintain salt and energy homeostasis. The abnormal growth of adrenocortical cells leads to an adrenocortical tumor (ACT). ACT is a rare cancer mainly caused by germline mutation in TP53, with an annual incidence of about 0.3 million in children below 15 years old [1,2]. Correlated with its physiological function, in most cases, ACT is associated with virilizing features and hypercortisolism. To date, the molecular pathogenesis of ACT has not been well studied; in most cases, the overexpression of insulin-like growth factor 2 and steroidogenic factor 1 were observed in ACT [3,4]. The constitutive activation of Wnt/β-catenin signaling also contributes to the development of ACT [5]. In the clinic, treatment of ACT mainly depends on surgical removal and chemotoxic therapies, and several chemotherapeutic drugs are used widely, including doxorubicin, cisplatin, mitotane, and etoposide [6].

Etoposide (ETO) has been used for a long period of time to treat ACT [7]. ETO is a topoisomerase II inhibitor that induces DNA double-strand breaks, thereby triggering apoptosis in cancer cells [8,9]. Etoposide induces the signaling of several phosphatidylinositol 3-kinase-related kinases (PI3KKs). Activated PI3KKs include ataxia-telangiectasia mutated (ATM), ataxia telangiectasia and Rad3-related protein (ATR), and DNA-dependent protein kinase (DNA-PK) trigger downstream effectors, thereby promoting cell cycle arrest, DNA repair, and even apoptosis [10,11]. Our previous study showed that ETO treatment induces senescence in ACT. However, whether PI3KK-mediated signaling plays a role in ETO-induced cellular events remains unclear.

The centrosome is a non-membranous organelle that functions as the primary microtubule organizing center [12]. During interphase, the centrosome orchestrates microtubule networks to maintain cell polarity, migration, and shape. The centrosome also nucleates mitotic spindles to segregate replicated chromosomes equally during mitosis [12,13,14]. The centrosome is surrounded by pericentriolar material (PCM) with two centrioles: mother and daughter centrioles [12]. Centrosome duplication is coordinated with DNA replication [15]. When cells enter the S phase, the centrosome starts to duplicate. Each parental centriole functions as a platform for a new centriole to grow in the orthogonal relationship. The new centriole becomes disengaged from the parental centriole during mitosis, thus ensuring centriole duplication in the next cell cycle [16]. Aberrant centriole disengagement leads to multiple centrosomes, and each PCM contains a single centriole [17]. Multiple centrosomes might lead to aberrant mitotic spindles. Thus, precise control of centrosome numbers is important to maintaining genomic integrity [10,18,19].

When cells enter the quiescent phase, the mother centriole serves as the basal body for primary cilia to grow [20,21]. The primary cilium is composed of the central acetylated microtubule known as the axoneme and the surrounding ciliary membrane [22]. In addition, several intraflagellar transporters are shuttled on the axoneme to regulate the length of the primary cilia [23]. Several signaling receptors, such as the G protein-coupled receptor, hedgehog, and PDGFRα, reside on the primary cilia. Thus, this antenna-like organelle is required for proper development and differentiation. In addition to the G0 quiescent phase, genotoxic stresses also facilitate primary cilia formation [24], and aberrant primary cilia also contribute to cellular senescence [25].

Autophagy was originally defined as a catabolic process that maintains metabolic homeostasis by degrading macro-molecules or organelles into essential subunits for recycling [26]. During starvation, the phagophore—the precursor of the autophagosome—is formed by converting LC3 I to LC3 II via ATG7-mediated lipidation [27]. Mature autophagosome-enclosed degradation targets then fuse with the lysosome and become autolysosomes to degrade the inner contents. In addition to its canonical role in maintaining energy homeostasis, autophagy also contributes to primary cilia formation [28]. Upon serum starvation, OFD1, a centriolar satellite protein, is degraded by autophagy followed by recruiting Bardet–Biedl syndrome 4 (BBS4) to the cilia for primary ciliogenesis [29]. Thus, autophagy plays multiple roles in regulating cellular physiology.

The molecular mechanism by which ETO inhibits tumor cell growth has been studied extensively. Our previous study showed that ETO induces centrosome amplification for cellular senescence in adrenocortical tumor cells. Here we demonstrated that primary cilia also play a role in ETO-induced cellular senescence. In addition, ETO activates DNA-PK-Chk2 axis for inducing autophagy, thereby modulating centrosomal and ciliary events. Thus, our study links PI3KK signaling to autophagy and primary cilia in adrenocortical tumors upon ETO treatment.

## 2. Materials and Methods

### 2.1. Cell Culture and Drug Treatment

We cultured mouse adrenocortical tumor Y1 cells in Dulbecco’s modified Eagle medium (DMEM)-F12 medium and human embryonal kidney cells transformed with SV40 large T antigen 293FT cells in DMEM at 37 °C in a humidified atmosphere at 5% CO_2_. All media were supplemented with 10% fetal bovine serum. DAPI staining was regularly used to check whether the Y1 and 293FT cells were free of mycoplasma contamination.

For drug treatment, cells were incubated with or without vanillin (DNA-PK inhibitor, V110-4, 1 mM), etoposide (ETO, E1383, at 10, 20, 50, 100, or 200 μM), roscovitine (centrosome inhibitor, R7772, 20 μM), Ku55933 (competitive ATM kinase inhibitor, SML1109, 10 μM), 3-methyladenine (3-MA, autophagy inhibitor, 5142-23-4, 5 mM), UCN-01 (Chk1 selective inhibitor, U6508, 100 nM), and chloroquine (CQ, autophagy inhibitor, 50-63-5, 50 μM), which were purchased from Sigma, St. Louis, MO, USA. Chk2 inhibitor II (Chk2 selective inhibitor, 220,491, 10 μM) was purchased from Merck Millipore, Darmstadt, Germany. Akt inhibitor IV (Akt selective inhibitor, 124,011, 5 µM) was purchased from Cell Signaling, Beverly, MA, USA. Cells were treated with drugs for 24 h, except those treated with ETO were treated for 24, 48, or 72 h.

### 2.2. Flow Cytometry

Each cell cycle stage was acquired and analyzed by fluorescence-activated cell sorting according to our published methodology [10]. In brief, cells were trypsinized and re-suspended with PBS-E (1 mM EDTA in PBS). Then, the cells were centrifuged at 1000 rpm for 5 min. After removing the supernatant, the pellets were re-suspended with PBS-E. After the 2nd centrifugation, the cells were re-suspended with 0.5 mL PBS-E and fixed with 4.5 mL 70% ice-cold ethanol at 4 °C for 18 h. Before analysis, the cells were extensively washed with PBS-E followed by staining with propidium iodide (PI, Southern Biotech, Birmingham, AL, USA) for 1 h at room temperature. DNA contents were measured and collected by FACScan (Becton-Dickinson, San Diego, CA, USA). All data were analyzed using the Kaluza software (Beckman Coulter, Brea, CA, USA).

### 2.3. EdU Incorporation Assay

The EdU incorporation assay was performed according to the user’s instructions (Invitrogen, Carlsbad, CA, USA). Cells were cultured with EdU for 8 h followed by fixation with fixing reagent provided by the EdU incorporation assay kit. To visualize the nuclei, the cells were double-stained with DAPI to count the EdU-positive cells.

### 2.4. Immunofluorescence Microscopy

Cells were grown on coverslips at 37 °C before drug treatment. After drug treatment, the cells were fixed with ice-cold methanol at −20 °C for 6 min. The tested cells were then blocked with 1% normal goat serum (NGS) for 1 h. After 1 h NGS blocking, the cells were incubated with specific antibodies. Next, 18 h after incubating with the first antibodies, the cells were washed with PBST and incubated with fluorescein isothiocyanate-conjugated and Cy3-conjugated secondary antibodies (Invitrogen, Carlsbad, CA, USA) for an additional 1 h. The DNA were simultaneously co-stained with 4′,6-diamino-2-phenylindole (DAPI, 0.1 μg/mL). After 1 h of incubation with secondary antibodies, the cells were washed with PBST, and the coverslips were mounted in 50% glycerol/PBST on glass slides. The fluorescence signals were examined with an AxioImager M2 fluorescence microscope (Zeiss, Switzerland).

### 2.5. Western Blotting Assay

After drug treatment, the cells were washed with PBS and further lysed with a lysis buffer containing 0.5% NP-40, 300 mM NaCl, 1 mM EDTA, and a protease inhibitor cocktail (Roche, Mannhein, Germany). The supernatant was centrifuged (15,000 rpm, 4 °C) and collected for further analysis by Western blotting. The relative intensity of region of interest was quantified by Image J (Java-based image-processing and analysis software). Pixels of interested protein were divided by internal control in each individual lane. Then, relative intensity was obtained by normalizing each experimental group to the control group. All data are presented as the mean ± S.D. from three independent experiments. Unpaired two-tailed *t*-tests were used to show the differences between two groups. *p*-values of less than 0.05 were considered statistically significant.

### 2.6. Antibodies

Antibodies used in this study were shown in the Table 1.

### 2.7. RNA Interference (RNAi)

*IFT88* was depleted in Y1 cells using lentivirus-containing plasmids expressing shRNA:

pLKO.1-shluc (5′-CCUAAGGUUAAGUCGCCCUCG-3′)

pLKO.1-shIFT88 (B3): 5′- GCAGGAAGACUGAAAGUGAAU [dt] [dt]-3′

pLKO.1-shIFT88 (C3): 5′- GCCCUCAGAUAGAAAGACCAA [dt] [dt]-3′

Lentiviruses were generated by transfecting 293FT cells with pLKO.1-shRNA plasmids, pCMVdelR8.91 (packaging vectors), and pMD.G (packaging vectors) according to the protocol provided by the National RNAi Core Facility, Taipei, Taiwan.

### 2.8. Mitotic Index

Mitotic cells were counted according to our published protocol [30]. In brief, mitotic cells, including cells in the pro-, prometa, meta-, ana-, and telo-phase, were counted in all populations of cells. At least 1000 cells were counted in each independent experiment.

### 2.9. Statistical Analysis

All results are expressed as the mean +/− S.D. from at least three independent experiments, and more than 100 cells were counted in each individual group. Differences between two groups were compared using unpaired two-tailed *t*-tests or one-way analysis of variance (ANOVA) tests, combined with the Tukey’s multiple comparison test for posterior comparisons. A *p*-value of less than 0.05 was considered to be statistically significant.

## 3. Results

### 3.1. Etoposide Inhibits Adrenocortical Tumor Cell Growth

**Figure 1:**
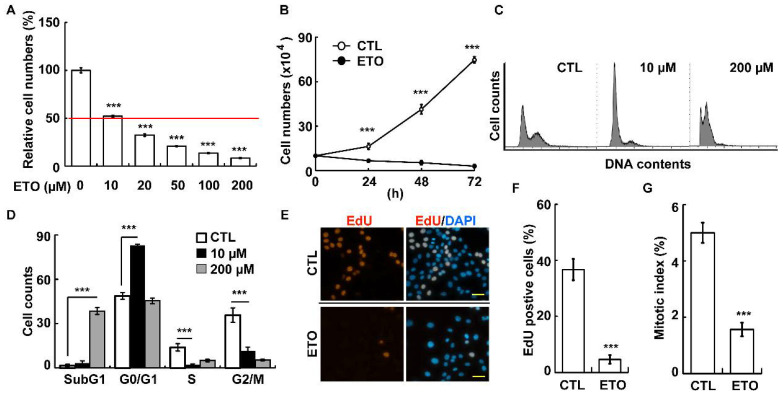
ETO inhibited Y1 cell proliferation. (**A**,**B**) ETO reduced Y1 cell numbers in a dose- and time-dependent manner.. (**A**) Cells were treated with different concentrations of ETO for 24 h followed by counting the cell numbers. (**B**) Y1 cells were treated with 10 μM ETO for different time periods. (CTL: control). (**C**,**D**) Cell cycle profiles are shown according to the flow cytometry analysis. Y1 cells were treated with 10 or 200 μM ETO followed by analysis with flow cytometry. (**E**,**F**) ETO inhibited S-phase entry. (**E**) The ability of cells to enter the S phase was analyzed using an EdU incorporation assay. (**F**) Quantitative results of (**E**). (**G**) ETO inhibited M-phase entry. The ability of cells to enter the M phase was assessed using the mitotic index. *** *p* < 0.001.

Etoposide (ETO) was previously used for treating adrenocortical cancers [7]. Our previous study showed that a sublethal dose (10 or 100 μM) of ETO treatment for 72 h induces G2/M arrest without triggering cellular apoptosis in ACT cells [31]. To more deeply investigate the molecular mechanism by which ETO inhibited ACT growth, we established the half maximal inhibitory concentration (IC50) of ETO on adrenocortical tumor Y1 cells. Adrenocortical tumor Y1 cells were treated with etoposide at different concentrations for 24 h, and the Y1 cell numbers reduced significantly in a dose- and time-dependent manner (Figure 1A,B). After 24 h of drug treatment, the IC50 of ETO on Y1 cells was observed to be 10 μM. This experimental condition was used in the following experiments. Previously, we showed that 72 h treatment of ETO at 10 μM led to cell cycle arrest [31]; we then examined the cell cycle profiles according to our experimental conditions. When Y1 cells were treated with 10 μM ETO for 24 h, an increased G0/G1 (2N) peak was observed (Figure 1C,D) without increasing the proportion of sub-G1 (apoptotic) cells. However, when Y1 cells were treated with 200 μM ETO, robust cell death was observed, as shown by the elevated sub-G1 cells. To further confirm 10 μM ETO treatment after 24 h of induced G0/G1 arrest, the ability of the cells to enter the S phase was examined using an EdU incorporation assay. Twenty-four hours after 10 μM ETO treatment, the proportion of EdU positive cells reduced significantly compared to that in the control group (Figure 1E,F). In addition, a lower mitotic index was observed, indicating that ETO impeded M-phase entry (Figure 1G). These data suggest that treatment of 10 μM ETO for 24 h impeded Y1 cell-cycle progression.

### 3.2. Etoposide Induces Cellular Senescence in Adrenocortical Tumor Cells

**Figure 2:**
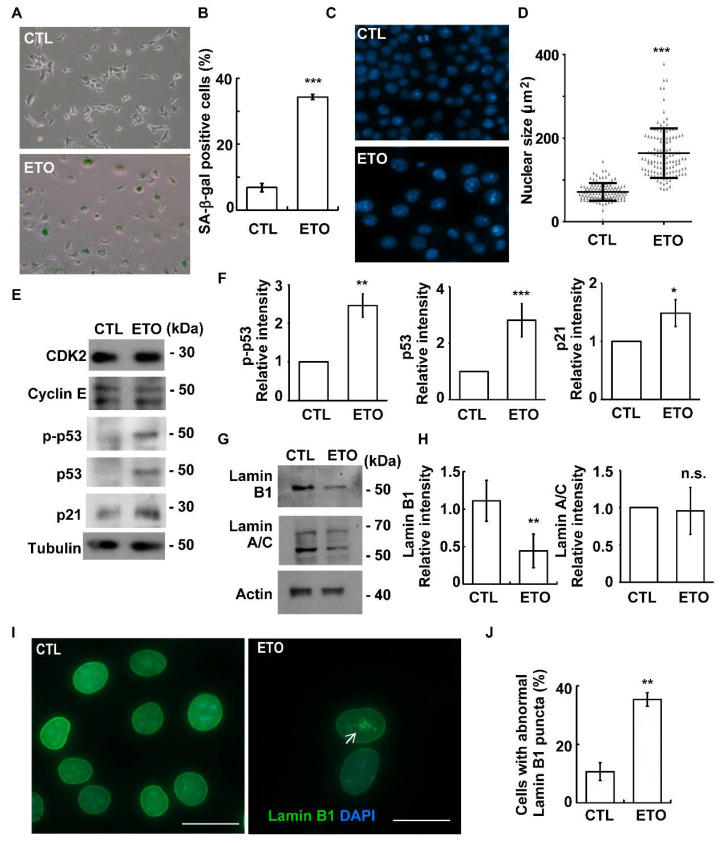
ETO induces cellular senescence in Y1 cells. (**A**,**B**) ETO-induced cellular senescence. Senescence-associated β-galactosidase (SA-β-gal) activity is shown (**A**, dark green in the cytoplasm, lower panel) and quantified (**B**) in the absence (CTL) or presence of ETO. (**C**,**D**) ETO treatment led to nuclei enlargement. (**C**) Nuclei were stained with DAPI in the absence or presence of ETO. (**D**) Quantification of nuclear size in the control or ETO-treated Y1 cells. (**E**,**F**) ETO activated the p53-p21 axis. Extracts of ETO-treated Y1 cells were analyzed using immunoblotting assay with antibodies against CDK2, cyclin E, phosphorylated p53 at Ser15 (p-p53), p53, p21, and tubulin. (**F**) Quantitative results of p-p53, p53, and p21 in (E). (**G**,**H**) ETO treatment reduced the level of Lamin B1. (**G**) Extracts of ETO-treated Y1 cells were analyzed with an immunoblotting assay using antibodies against Lamin B1, Lamin A/C, and actin. (**H**) Quantitative results of Lamin B1 and Lamin A/C (**G**). (**I**,**J**) Nuclear Lamin B1 puncta were formed upon ETO treatment. (**I**) Immunofluorescence staining of cells with antibodies against Lamin B1 in the control or ETO-treated Y1 cells. DNA was stained with DAPI. (**J**) Quantification results for cells with nuclear Lamin B1 puncta formation (**I**). n.s.: no significance; * *p <* 0.05; ** *p <* 0.01; *** *p <* 0.001.

Our previous study showed that treatment of ETO for 72 h induced cellular senescence, as evidenced by increasing senescence-associated β-galactosidase (SA-β-gal) activity and enlarged nuclei [31]. We thus tested whether treatment with 10 μM ETO for 24 h was sufficient to trigger senescence. The activity of SA-β-gal was also examined. Normally, the activity of SA-β-gal is difficult to detect; however, upon 24 h ETO treatment, the activity of SA-β-gal increased dramatically (Figure 2A,B). In addition, enlarged nuclei were observed (Figure 2C,D). These data suggest that treatment with 10 μM ETO for 24 h is sufficient to induce cellular senescence. Moreover, the activated p53-p21 axis induced cellular senescence [32,33]. To further confirm our findings, the p53-p21 axis was examined. Upon ETO treatment for 24 h, the levels phosphorylated and the total p53 increased (Figure 2E,F). Moreover, p21 was upregulated (Figure 2E,F), indicating that 24 h ETO treatment induced senescence. Furthermore, the level of Lamin B1 was examined, as lower Lamin B1 leads to cellular senescence [34]. The abundance of Lamin A/C was not affected. However, ETO treatment for 24 h was observed to reduce Lamin B1 (Figure 2G,H). Interestingly, when examining Lamin B1 by immunofluorescence staining, we observed that ETO treatment not only reduced the intensity of Lamin B1 but also led to abnormal accumulations of Lamin B1 puncta in the nucleus (Figure 2I,J). Thus, the data indicate that treatment with 10 μM ETO for 24 h is sufficient to induce cellular senescence.

### 3.3. Etoposide Promotes Cellular Senescence by Inducing Centrosome Amplification

**Figure 3:**
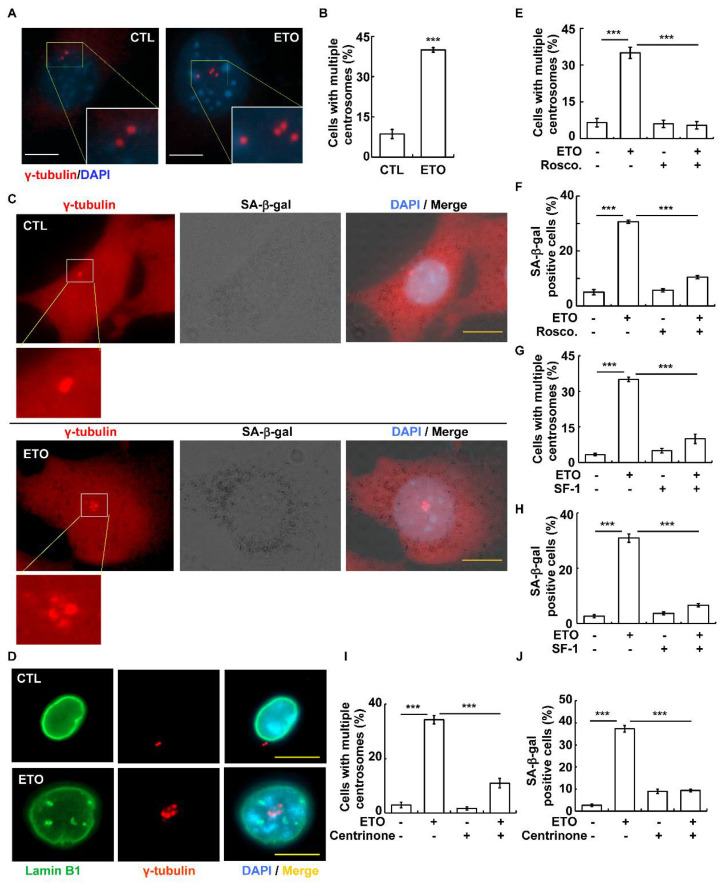
ETO induced cellular senescence by triggering multiple centrosomes. (**A**,**B**) ETO induced multiple centrosomes (more than 2 centrosomes). (**A**) Immunofluorescence staining of cells with the antibody against γ-tubulin. DNA was stained with DAPI. Scale bar 10 μm. (**B**) Quantification results of cells with multiple centrosomes as shown in (**A**). (**C**) Y1 cells with multiple centrosomes were senescent upon ETO treatment. After ETO treatment for 24 h, cells were stained with SA-β-gal kit. After extensive washing and fixation, cells were blocked with 3% normal goat serum followed by immunofluorescence staining with antibody against γ-tubulin. (**D**–**J**) Multiple centrosomes contributed to ETO-induced senescence. (**D**) Cells with multiple centrosomes were senescent cells. (**E**,**F**) Inhibition of multiple centrosomes alleviated ETO-induced senescence. (**E**) Treatment of cells with roscovitine (Rosco.) inhibited multiple centrosomes. (**F**) Roscovitine alleviated ETO-induced cellular senescence. (**G,H**) Overexpression of NR5A1/SF-1 alleviated ETO-induced senescence. Overexpression of NR5A1/SF-1 in Y1 cells inhibited (**G**) multiple centrosomes and (**H**) cellular senescence. (**I,J**) Inhibition of multiple centrosomes by treating cells with centrinone alleviated ETO-induced senescence. Treating cells with centrinone inhibited (**I**) multiple centrosomes and (**J**) cellular senescence. *** *p <* 0.001.

Our previous study showed that multiple centrosomes lead to senescence without triggering DNA damage in adrenocortical tumors [19]. We thus examined whether ETO-induced senescence was caused by multiple centrosomes. Normally, cells contain one (before duplication) or two centrosomes (after duplication), as shown by γ-tubulin staining; however, cells containing multiple centrosomes (cells with more than two centrosomes) were observed in ETO-treated Y1 cells (Figure 3A,B) in a time-dependent manner (Figure S1A). Then, we examined whether the cells harboring multiple centrosomes were senescent cells. Y1 cells were pre-stained with an SA-β-gal kit followed by staining the centrosome with an antibody against γ-tubulin. After SA-β-gal staining, a γ-tubulin signal with a lower signal-to-noise ratio could be detected by immunofluorescence staining, and more than 90% of the cells with multiple centrosomes were senescent cells (Figure 3C). To further confirm this finding, cells were co-stained with centrosome and Lamin B1. Intact Lamin B1 was clearly detected in the perinuclear region (Figure 3D, upper panel). Upon ETO treatment, multiple centrosomes were observed, and most of the cells with multiple centrosomes showed reduced perinuclear Lamin B1 with nuclear Lamin B1 puncta (Figure 3D, lower panel). The data indicate that these multiple centrosome-harboring cells were senescent cells.

Next, we assessed whether the ETO-induced multiple centrosomes facilitated cellular senescence. Roscovitine is a known inhibitor that blocks multiple centrosomes efficiently [35]. (Figure 3D) Treatment with roscovitine alleviated the ETO-induced multiple centrosomes (Figure 3E). Cellular senescence was also reduced in roscovitine-treated cells (Figure 3F), suggesting that these multiple centrosomes contributed to senescence when Y1 cells were treated with 10 μM ETO for 24 h. Overexpression of NR5A1/SF-1 inhibited multiple centrosomes during prolonged replication stress in adrenocortical tumor cells [36]. Further confirming that the ETO-induced multiple centrosomes contributed to senescence, ectopic SF-1 was expressed in Y1 cells. The ETO-induced multiple centrosomes were inhibited by SF-1 overexpression (Figure 3G). Cellular senescence was also reduced in SF-1-overexpressing Y1 cells (Figure 3H). Moreover, multiple centrosomes were inhibited by treating cells with centrinone, a PLK4 inhibitor that blocks centrosome amplification [37]. Centrinone inhibited ETO-induced multiple centrosomes (Figure 3I) and also senescence (Figure 3J). Thus, ETO induced multiple centrosomes, thereby leading to cellular senescence.

**Figure 4:**
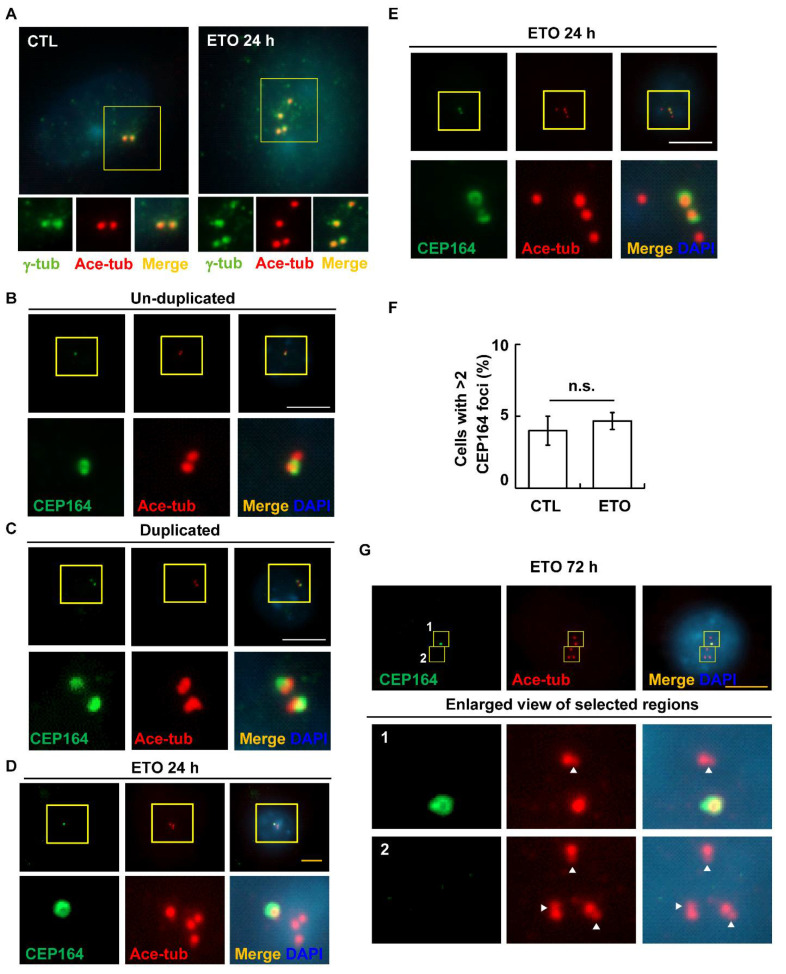
ETO induces centriole disengagement. (**A**) ETO treatment for 24 h resulted in centriole disengagement. Immunofluorescence staining of cells with antibodies against γ-tubulin (γ-tub, marker of PCM) and acetylated tubulin (Ace-tub, marker of centriole). (**B**–**F**) ETO treatment for 24 h did not induce multiple CEP164 foci. The mother centriole (CEP164) was examined by immunofluorescence staining with antibodies against CEP164 and acetylated tubulin before (**B**) or after centriole duplication (**C**) in the absence (**B**,**C**) or presence of ETO for 24 h (**D**,**E**). (**F**) Quantification results of cells with more than two CEP164 foci in the absence or presence of ETO for 24 h. (**G**) Centriole duplication occurred upon ETO treatment for 72 h. Lower panels provide enlarged views of the selected regions shown in the upper panel. White arrowheads indicate newly growing centrioles after centriole disengagement. DNA was stained with DAPI. Scale bar: 10 μm.

Multiple centrosomes may be caused by PCM fragmentation or by centriole disengagement [38,39]. In addition, the radiomimetic drug doxorubicin was shown to induce centriole disengagement in pigmented epithelial retina cells [40]. Aberrant centriole disengagement leads to multiple centrosomes, and each PCM contained a single centriole [17]. We thus examined these amplified centrosomes using the double-staining of cells with markers of PCM (γ-tubulin) and centrioles (acetylated tubulin). After ETO treatment for 24 h, each PCM was found to contain one centriole (Figure 4A), indicating that the ETO-induced multiple centrosomes were not caused by PCM fragmentation but by centriole disengagement. Centriole disengagement was further examined. Before centrosome duplication, cells contained a mother centriole (CEP164 positive centriole) and a daughter centriole (ETO centriole without CEP164) (Figure 4B). After centrosome duplication, four centrioles were observed and only two of them were mother centrioles (Figure 4C). Following ETO treatment for 24 h, four separated centrioles were observed, and one or two out of four centrioles were mother centrioles (Figure 4D,E). The proportion of cells with more than two CEP164 signals did not increase (Figure 4F), suggesting that ETO led to centriole disengagement in Y1 cells. Centriole disengagement leads to centriole re-duplication [18]. We then examined the effect of long-term centriole disengagement on centriole copy numbers in Y1 cells. Upon ETO treatment for 72 h, cells with more than four centrioles were observed (Figure 4G). Interestingly, these preexisting centrioles started to grow new centrioles (Figure 4G, lower panels, white arrowhead). Thus, the data show that ETO treatment leads to centriole disengagement.

### 3.4. ETO Induces Primary Cilia Formation

**Figure 5:**
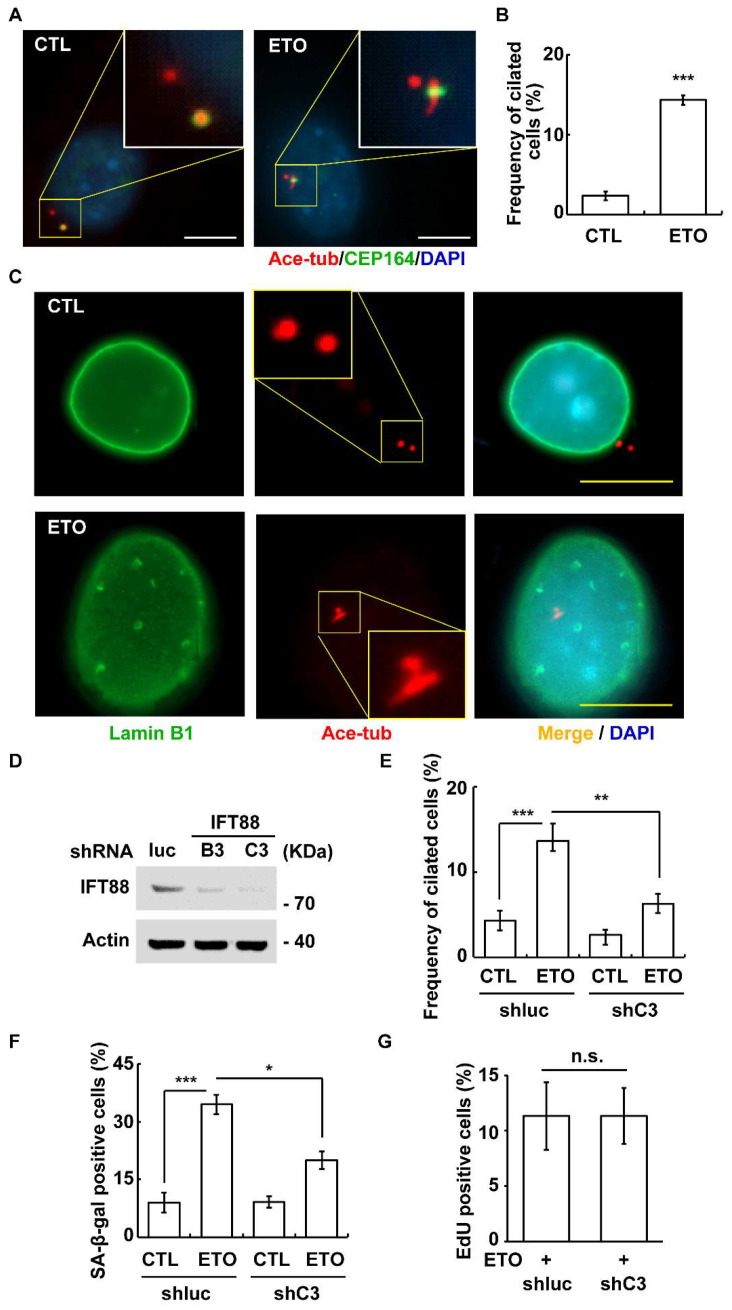
ETO-induced primary cilia facilitated cellular senescence. (**A**,**B**) ETO induced primary cilia formation. (**A**) Immunofluorescence staining of ETO-treated cells with antibodies against CEP164 and acetylated tubulin (Ace-tub). (**B**) Quantification results of the population of ciliated cells in (**A**). (**C**) Ciliated cells were senescent cells. Immunostaining of cells with antibodies against Ace-tub and Lamin B1 in the absence (CTL) or presence of ETO. (**D**–**G**) Disruption of primary cilia alleviated ETO-induced cellular senescence. (**D**) IFT88 was depleted efficiently. Extracts of lentivirus (containing shRNA against luc or IFT88 B3 and C3)-infected Y1 cells were analyzed by immunoblotting with antibodies against IFT88 and actin. (**E**) Depletion of IFT88 inhibited primary cilia formation. Quantitative results of the proportion of ciliated cells in the control (shluc) and IFT88-depleted Y1 cells (shC3). (**F**) Depletion of IFT88 alleviated ETO-induced cellular senescence. Quantitative results of the proportion of senescent cells in shluc and shC3 Y1 cells. (**G**) Depletion of IFT88 did not facilitate cells entering the S phase upon ETO treatment as shown by EdU incorporation. n.s.: no significance; * *p <* 0.05; ** *p <* 0.01; *** *p <* 0.001.

Primary cilia initiate senescence [41], and inhibition of the primary cilia attenuates premature senescence in human diploid fibroblasts [25]. We thus examined the primary cilia in ETO-treated adrenocortical tumor cells. ETO induced multiple centrosomes (Figure 3A). However, some ETO-treated Y1 cells did not form multiple centrosomes but instead started to grow primary cilia (Figure 5A,B). We thus examined whether ciliated cells were senescent cells by staining cells with primary cilia and Lamin B1. Intact Lamin B1 was clearly detected in the perinuclear region (Figure 5C, upper panel). However, upon ETO treatment, cells started to grow primary cilia, and these ciliated cells showed reduced Lamin B1 with nuclear Lamin B1 puncta (Figure 5C, lower panel). Thus, the data imply that primary cilia might contribute to cellular senescence. We then tested whether ETO-induced primary cilia contributed to cellular senescence in ACT cells. IFT88, an intraflagellar transporter that maintains primary cilia, was depleted by infecting Y1 cells with lentivirus containing shRNA against IFT88. Two shRNA sequences, B3 and C3, were tested, and both B3 and C3 inhibited IFT88 expression efficiency (Figure 5D). The depletion of IFT88 inhibited ETO-induced primary cilia formation in Y1 cells (Figure 5E). We then examined whether primary cilia contributed to ETO-induced senescence. Upon 10 μM ETO treatment for 24 h, the proportion of senescent cells increased. However, this phenotype was alleviated in IFT88-deficient cells (Figure 5F), suggesting that the primary cilia contributed to ETO-induced senescence. To rule out the possibility that the reduced senescence in IFT88-depleted cells was caused by cells escaping from G0/G1 arrest, the ability of cells to enter the S phase was examined. As shown by the EdU-incorporation assay, depletion of IFT88 did not facilitate cells entering the S phase upon ETO treatment (Figure 5G). Thus, consistent with previous findings in fibroblast cells, ETO-induced primary cilia also contributed to cellular senescence without affecting G0/G1 arrest in adrenocortical tumor cells.

### 3.5. DNA-PK-Chk2 Axis Triggers Multiple Centrosomes and Primary Cilia

**Figure 6:**
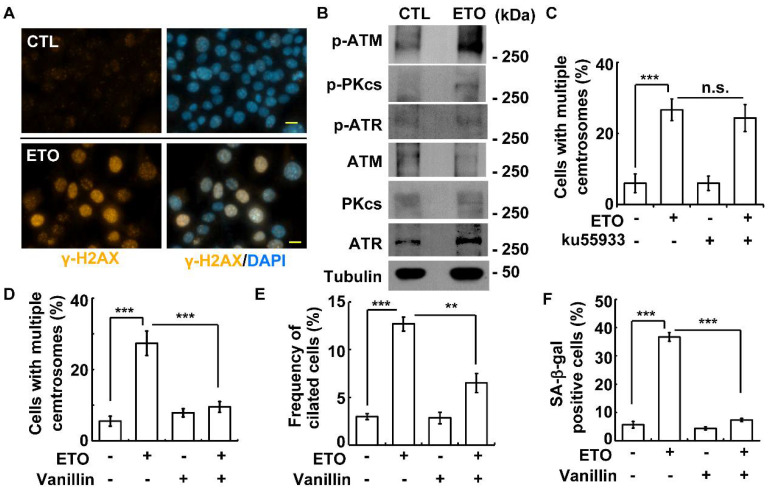
ETO-activated DNA-PK induced centrosome amplification, primary cilia formation, and cellular senescence. (**A**) ETO induced DNA damage. The DNA damage marker γ-H2AX was shown by immunofluorescence staining with the antibody against γ-H2AX. DNA was stained with DAPI. (**B**) ATM and DNA-PK were activated by ETO. Extracts of ETO-treated Y1 cells were analyzed by an immunoblotting assay with antibodies against ATM, phosphorylated ATM (p-ATM), ATR, phosphorylated ATR (p-ATR), DNA-PKcs (PKcs), phosphorylated DNA-PKcs (p-PKcs), and tubulin. (**C**) Inhibition of ATM did not affect centrosome amplification. Quantitative results of cells with multiple centrosomes in the presence or absence of the ATM inhibitor (Ku55933). (**D**–**F**) Inhibition of DNA-PK alleviated centrosome amplification, primary cilia formation, and cellular senescence. Quantitative results of the proportions of cells with multiple centrosomes (**D**), primary cilia (**E**), and positive SA-β-gal staining (**F**) in the absence or presence of vanillin (DNA-PK inhibitor). n.s.: no significance; ** *p <* 0.01; *** *p <* 0.001.

Irradiation and prolonged replication stress induce multiple centrosomes in osteosarcoma U2-OS cells via DNA damage responses [10,42]. Etoposide induces DNA damage and phosphatidylinositol 3-kinase-related kinase (PI3KK) signaling [10,11]. Our previous study showed that ETO treatment induces senescence in ACT. However, whether PI3KK-mediated signaling plays a role in ETO-induced cellular events remains unclear. We thus assessed whether ETO induced multiple centrosomes or ciliogenesis via PI3KKs in ACT cells. As published in [31], ETO induced DNA damage, which was shown by γ-H2AX staining in the Y1 cells (Figure 6A). We then examined whether ETO activated PI3KKs in adrenocortical tumors. ATM and DNA-PK, but not ATR, were activated by ETO treatment (Figure 6B). The inhibition of ATM by its selective inhibitor ku55933 [43] had no effect on ETO-induced multiple centrosomes (Figure 6C). However, the inhibition of DNA-PK by treating cells with vanillin [44] alleviated both ETO-induced multiple centrosomes and primary cilia (Figure 6D,E). In addition, the inactivation of DNA-PK also reduced ETO-induced cellular senescence (Figure 6F). These data suggest that ETO-activated DNA-PK facilitates cellular senescence, at least in part, via multiple centrosomes and primary cilia.

**Figure 7:**
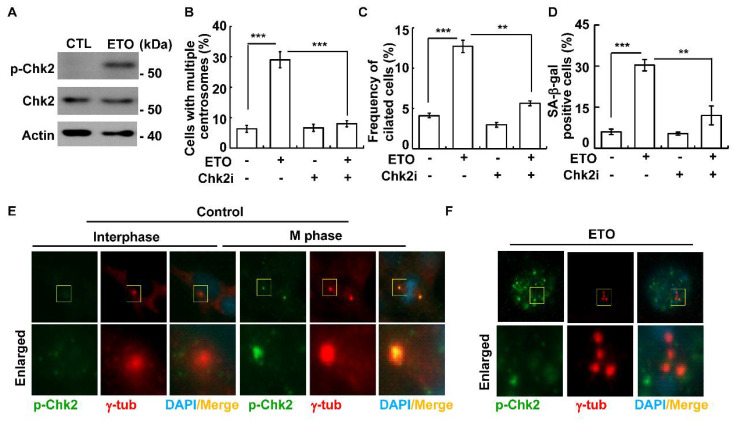
Chk2 induced centrosome amplification, primary cilia formation, and cellular senescence in ETO-treated Y1 cells. (**A**) ETO-activated Chk2. Extracts of ETO-treated activated Chk2. Extracts of ETO-treated Y1 cells were analyzed by immunoblotting with antibodies against phosphorylated Chk2 (p-Chk2), Chk2, or actin. (**B**–**D**) Inhibition of Chk2 alleviated centrosome amplification, primary cilia formation, and cellular senescence. Quantitative results of the proportions of cells with multiple centrosomes (**B**), primary cilia (**C**), and positive SA-β-gal staining (**D**) in the absence or presence of the Chk2 inhibitor (Chk2i). (**E**,**F**) Activated Chk2 was localized in the nucleus but not in the centrosome upon ETO treatment. Immunofluorescence staining of control cells in the interphase (**E**, left panel) and M phase (**E**, right panel), and ETO-treated cells (**F**) with antibodies against γ-tubulin and phosphorylated Chk2 (p-Chk2). DNA was stained with DAPI. ** *p <* 0.01; *** *p <* 0.001.

Then, the downstream effectors of DNA-PK, including AKT, Chk1, and Chk2, were examined. Both Chk1 and Chk2, but not AKT, were activated, as shown by their phosphorylations upon ETO treatment (Figure 7A and Figure S2A). The inactivation of Chk2, but not Chk1, with its selective inhibitor Chk2 inhibitor II alleviated ETO-induced multiple centrosomes and primary cilia (Figure 7B,C and Figure S2B). In addition, the inhibition of Chk2 also reduced ETO-induced cellular senescence (Figure 7D). The data suggest that ETO-activated Chk2 contributes to senescence, at least in part, by triggering primary cilia and multiple centrosomes.

Activated Chk2 is not only localized to the nucleus but also to the centrosome in osteosarcoma U2-OS cells [10]. We thus assessed the subcellular localization of activated Chk2 in Y1 cells upon ETO treatment. During interphase, Chk2 was difficult to detect (Figure 7E, left panel). However, when cells entered the M phase, phosphorylated Chk2 was enriched in the mitotic spindle poles (Figure 7E, right panel). These data are consistent with the previous finding that activated Chk2 localizes to mitotic spindle poles to maintain bipolar spindle formation [45]. Upon ETO treatment, phosphorylated Chk2 was mainly localized in the nuclei of Y1 cells. However, unlike the observations in osteosarcoma U2-OS cells, ETO-activated Chk2 was not detected in the centrosomes (Figure 7F). Thus, the primary cilia and multiple centrosomes might not be contributed by centrosomal Chk2 upon ETO treatment in Y1 cells.

### 3.6. ETO-Activated Autophagy Induces Multiple Centrosomes and Primary Cilia

**Figure 8:**
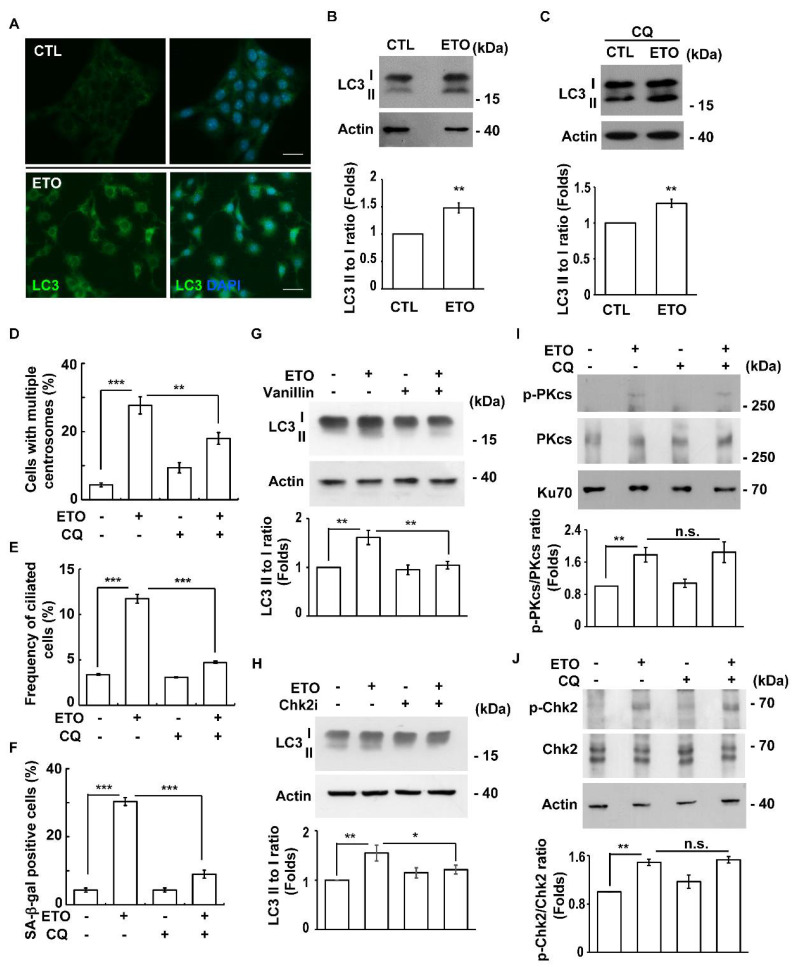
Autophagy contributes to centrosome amplification, primary cilia formation, and cellular senescence. (**A**–**C**) ETO-induced autophagy. (**A**) Immunofluorescence staining of ETO-treated Y1 cells with the antibody against LC3. (**B**,**C**) Extracts of Y1 cells treated with (**B**) ETO or (**C**) co-treated with ETO and chloroquine (CQ) were analyzed by immunoblotting with antibodies against LC3 and actin. Quantitative results of the LC3 II to I ratio are shown in the lower panel. (**D**–**F**) Chloroquine (CQ) alleviated centrosome amplification, primary cilia formation, and cellular senescence. Quantitative results of the proportions of cells with multiple centrosomes (**D**), primary cilia (**E**), and positive SA-β-gal staining (**F**) in the absence or presence of the autophagy inhibitor CQ. (**G**,**H**) Inhibition of DNA-PK (**G**) and Chk2 (**H**) alleviated ETO-induced autophagy. Extracts of ETO-treated Y1 cells in the presence of vanillin (**G**) or the Chk2 inhibitor (Chk2i) were analyzed by immunoblotting with antibodies against LC3 and Actin. (**I**,**J**) Inhibition of autophagy did not affect DNA-PK-Chk2 activation. Extracts of ETO-treated Y1 cells in the presence or absence of CQ were analyzed with immunoblotting using antibodies against phosphorylated DNA-PKcs (p-PKcs), DNA-PKcs (PKcs), phosphorylated Chk2 (p-Chk2), Chk2, Ku70, and actin. n.s.: no significance; * *p <* 0.05; ** *p <* 0.01; *** *p <* 0.001.

Autophagy regulates centrosome homeostasis and primary cilia formation [28,29,46]. Our previous study showed that ETO activates autophagy in Y1 cells [31]. We further tested whether ETO-activated autophagy participated in the multiple centrosomes and primary cilia. First, we confirmed that ETO induced autophagy. Using an immunofluorescence staining assay, the LC3 puncta were difficult to detect in the control cells (Figure 8A, upper panel). Upon ETO treatment, an accumulation of cytoplasmic LC3 puncta was observed (Figure 8A, lower panel), suggesting that autophagy was triggered. We then examined the autophagy flux by examining the conversion of LC3 I to II. The LC3 II to I ratio increased in the ETO-treated group compared to the control group, suggesting that autophagy was induced (Figure 8B). Furthermore, lysosomal degradation was blocked by treating the cells with chloroquine, a known lysosome inhibitor. A consistently increased LC3 II to I ratio was observed in the ETO-treated Y1 cells (Figure 8C). Thus, consistent with our previous findings, ETO induced autophagy in Y1 cells. We then examined whether ETO induced primary cilia formation and multiple centrosomes via autophagy. The inhibition of autophagy by treating cells with chloroquine and 3-MA alleviated ETO-induced multiple centrosomes and primary cilia (Figure 8D,E and Figure S4A). In addition, ETO-induced senescence was inhibited by blocking autophagy (Figure 8F and Figure S4B). Taken together, the results show that ETO induces autophagy for cellular senescence, and this process, at least in part, induces primary cilia and multiple centrosomes, thus leading to cellular senescence.

ETO activated the DNA-PK-Chk2 cascade and autophagy. Next, the causal relationship between these two signaling processes was examined. Inactivation of the DNA-PK-Chk2 cascade alleviated ETO-induced autophagy (Figure 8G,H). However, the inhibition of autophagy did not affect the activation of DNA-PK or Chk2 (Figure 8I,J). Thus, our data show that ETO activated the DNA-PK-Chk2 cascade for triggering autophagy.

## 4. Discussion

In this study, we determined the molecular mechanism by which ETO inhibits adrenocortical tumor growth. ETO triggered the DNA-PK-Chk2 signaling cascade for activating autophagy, followed by triggering adrenocortical Y1 cell senescence. Apart from its canonical functions, DNA-PK-Chk2-activated autophagy induced primary ciliogenesis and multiple centrosomes followed by senescence. Thus, our study reveals novel therapeutic effects of ETO on adrenocortical tumor cells.

In response to genomic stresses, DNA-PK is activated to maintain cell survival and repair damaged DNA [47]. In response to DNA damage, DNA-PK becomes activated to stabilize p53. The accumulation of p53 then induces cell cycle arrest and cellular senescence. In addition to regulating nuclear events, DNA-PK also induces centrosome amplification in steroidogenic cells when a key transcription factor nuclear receptor 5A1 (NR5A1) is depleted without triggering the DNA damage response [36]. In the absence of NR5A1, DNA-PK is activated only in the centrosome but not in the nucleus, thereby inducing centrosome amplification. A recent study also showed that DNA-PK induces primary cilia formation in normal pigmented epithelial retina cells under genotoxic stress [24]. Interestingly, in response to genotoxic stress, DNA-PK not only remains in the nucleus but also localizes to the centrosome, leading to primary cilia formation. In our study, we found that etoposide activates DNA-PK, leading to centrosome amplification and primary cilia formation. As nuclear and centrosomal DNA-PK contribute to centrosomal effects, we speculate that both nuclear and centrosomal DNA-PK are activated by ETO treatment. However, this hypothesis needs to be confirmed in the future.

DNA damage checkpoint kinases Chk1 and Chk2 participate in triggering multiple centrosomes [10,48,49]. Dodson et al. observed that DNA damage due to ionizing radiation induced supernumerary centrosomes, but not PCM fragmentation, in chicken lymphoma-B DT40 and human osteosarcoma U2OS cell lines. The depletion or inactivation of Chk1 alleviates DNA damage-induced supernumerary centrosomes, suggesting that Chk1 activity is required for amplifying the centrosomes [48]. In addition, the depletion of Rad51 recombinase induces spontaneous DNA damage and prolonged G2 phase arrest in chicken lymphoma-B DT40 cells. Meanwhile, multiple centrosomes are induced via ATM activation [49]. In our study, we found that, upon ETO treatment, the DNA-PK-Chk2 cascade was activated, thereby inducing multiple centrosomes in adrenocortical tumor cells. It remains unclear why our data are not consistent with the published results. One possibility is that previous studies mainly focused on irradiation- or Rad51-deficiency-induced DNA damage, which activated either Chk1 or ATM, leading to supernumerary centrosomes. However, in our study, we induced DNA damage by blocking the activity of topoisomerase II, thus activating DNA-PK-Chk2 signaling for DNA double-strand breaks. This signaling was also activated by prolonged replication stress, leading to centrosome amplification [10]. Thus, different types of DNA damage might trigger distinct types of signaling for multiple centrosomes. Second, previous studies used chicken lymphoma-B DT40 and human osteosarcoma U2OS cell lines to investigate centrosome homeostasis. In our study, we analyzed centrosome homeostasis by using mouse adrenocortical tumor Y1 cells. Distinct genetic backgrounds may trigger different signaling cascades, yielding multiple centrosomes.

Reduced Lamin B1 levels were observed in senescent cells [34]. Upon ETO treatment, the level of Lamin B1 was also reduced in Y1 cells. Upon examining these ETO-treated cells by immunofluorescence staining, several Lamin B1 puncta were observed in the nuclei. It remains unclear why Lamin B1 formed these puncta when its expression was reduced. Lamin B1 was subtracted via nuclear autophagy [50]. By forming a complex with LC3/ATG8, Lamin B1 was shuttled from the nucleus to the cytoplasm followed by autophagosome–lysosome degradation. This nuclear autophagy pathway was also used for the degradation of other nuclear protein NAD-dependent deacetylase sirtuin-1 (SIRT1) enzymes [51]. In this study, we showed that ETO induced autophagy in Y1 cells. We thus speculate that ETO-induced aggregations of nuclear Lamin B1 puncta served as the sites for forming LC3/ATG8 complexes followed by transportation from the nucleus to the cytoplasm for further autophagy–lysosome degradation. However, this hypothesis should be studied more extensively in the future.

Most mammalian cells can grow primary cilia in the G0 quiescent phase, during which these cilia function as cellular antenna for transducing signals in development and differentiation [20]. Thus, defective primary ciliogenesis leads to several diseases that are collectively called ciliopathy. Recent studies showed that a loss of primary cilia occurs during tumorigenesis in breast, lung, and pancreatic cancers [52]. Thus, most cancers are devoid of primary cilia. In this study, we showed that, upon ETO treatment, adrenocortical tumor Y1 cells started to grow primary cilia via DNA-PK-Chk2 signaling. Why Y1 cells grow primary cilia upon ETO treatment remains unclear. A recent study showed that lung cancer cells do not grow primary cilia; however, when these cells became drug-resistant, they regained the ability to grow cilia [53]. We thus speculate that, upon ETO treatment, Y1 cells start to grow cilia for cell cycle arrest and possibly, more importantly, gain the ability to become drug-resistant cells. This hypothesis should be further investigated in the future toward improving chemotherapeutic effects in the clinical setting.

## 5. Conclusions

In summary, we found that ETO activated the DNA-PK-Chk2 cascade for triggering autophagy, thus leading to cellular senescence. The underlying molecular mechanism might be contributed by primary cilia formation and multiple centrosomes. Ultimately, our study uncovered the novel mechanism by which ETO inhibited adrenocortical tumor growth.

## Figures and Tables

**Table 1:** All antibodies used in the study are listed below.

Antibodies	Company	Catalog Number
phospho-p53 (Ser15)	Cell signaling	#9284
p53	Santa Cruz	sc-6243
p21	Abcam	ab7960-1
actin (AC-15)	GeneTex	GTX26276
Lamin B1	Abcam	ab16048
Lamin A/C	Genetex	GTX101127
γ-tubulin	Sigma	T6557
tubulin-acetylated	Sigma	T6793
γ-H2A.X (Ser139)	Abcam	ab2893
ATM	GeneTex	GTX70103
phospho-ATM (Ser1981)	Abcam	ab81292
DNA-PKcs (H163)	Santa Cruz	sc-9051
phospho-DNA-PKcs (Thr2609)	Santa Cruz	sc-101664
ATR	Cell signaling	#2790
phospho-ATR	Cell signaling	#2853
Chk2	Cell signaling	#3440
phospho-Chk2 (Thr68)	Cell signaling	#2661
LC3 A/B	Cell signaling	#12741
CEP164	NOVUS	NBP1-81445
Phosphor-Chk1 (Ser317) (D12H3)	Cell signaling	#12302
Chk1 (2G1D5)	Cell signaling	#2360
Akt (C67E7)	Cell signaling	#4691
p-Akt (S473)	Cell signaling	#4060
α-tubulin	GeneTex	GTX112141

## Data Availability

Not applicable.

## Supplementary Materials

The following are available online at https://www.mdpi.com/article/10.3390/cells10061466/s1, Figure S1: ETO induces centrosome amplification. Figure S2: ETO-activated Chk1 does not contribute to centrosome amplification. Figure S3: Autophagy contributes to ETO-induced centrosome amplification and cellular senescence.
