# Supplementary material for: Etoposide Triggers Cellular Senescence by Inducing Multiple Centrosomes and Primary Cilia in Adrenocortical Tumor Cells
Source: Cells. 2021 Jun 11;10(6):1466. doi: 10.3390/cells10061466 (PMC8230646; doi:10.3390/cells10061466)
Supplement: Supplementary file 1 [file cells-10-01466-s001.zip › cells-1136765-supplementary.pdf]

Supplementary data

Supplementary Figure S1.

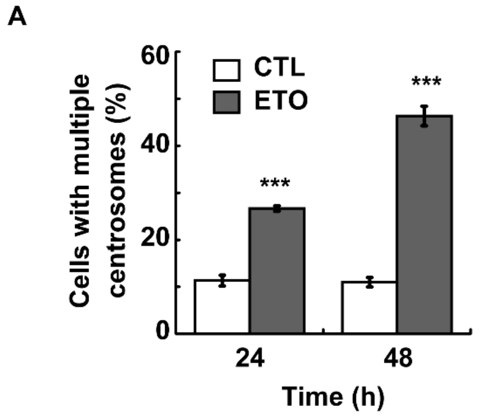

Supplementary Figure S1. ETO induces centrosome amplification.
(A) ETO induced centrosome amplification in a time-dependent manner. Quantitative results of cells with multiple centrosomes in the absence (CTL, upper panel) or presence of ETO (lower panel) for 24 or 48 h. ***: P<0.001.

Supplementary Figure S2.

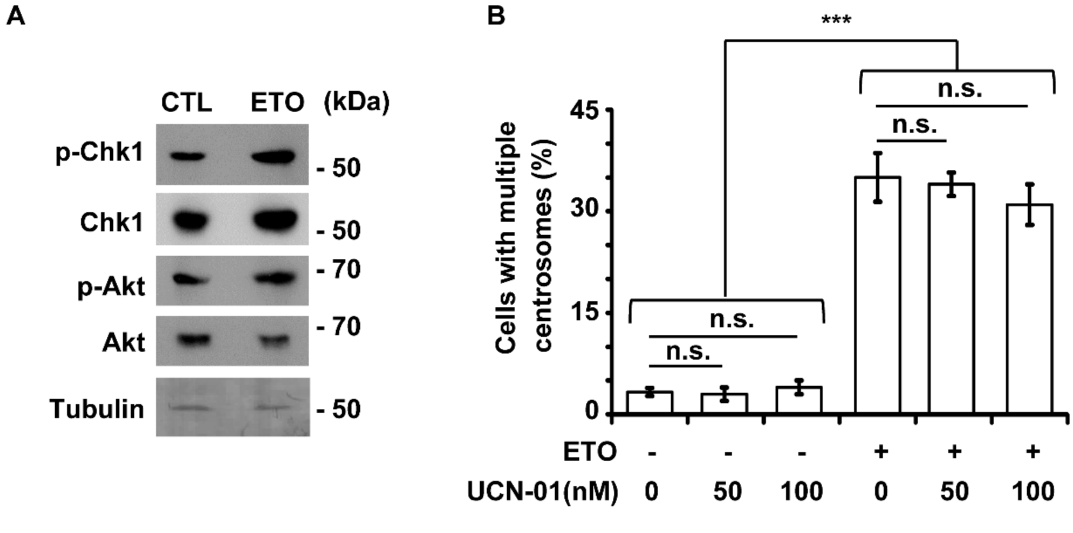

Supplementary Figure S2. ETO-activated Chk1 does not contribute to centrosome amplification. (A) ETO activated Chk1. Extracts of ETO-treated Y1 cells were analyzed by immunoblotting with antibodies against phosphorylated Chk1 (p-Chk1), Chk1, phosphorylated Akt (p-Akt), Akt, and tubulin. (B) Inactivation of Chk1 did not affected ETO-induced centrosome amplification. Quantitative results of cells with multiple centrosomes in the absence or presence of UCN-01 (Chk1 inhibitor) at different concentrations.

Supplementary Figure S3.

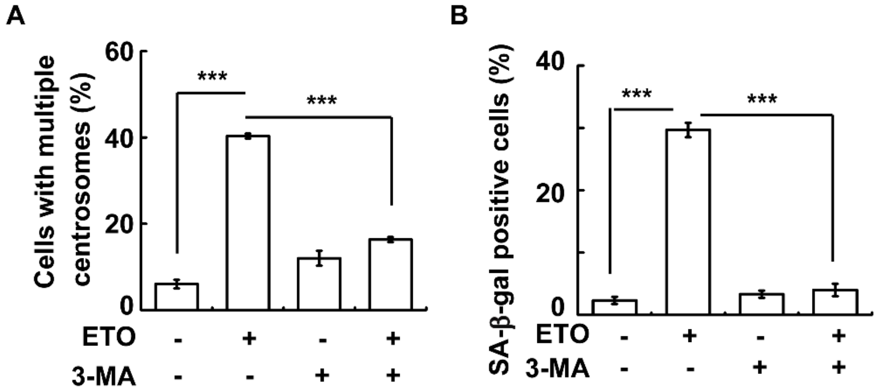

Supplementary Figure S3. Autophagy contributes to ETO-induced centrosome amplification and cellular senescence. (A-B) Quantitative results proportions of cells with multiple centrosomes (A) or with senescence (B) in the presence or absence of autophagy inhibitor (3-MA).
